# Superior Intrinsic Mitochondrial Respiration in Women Than in Men

**DOI:** 10.3389/fphys.2018.01133

**Published:** 2018-08-17

**Authors:** Daniele A. Cardinale, Filip J. Larsen, Tomas A. Schiffer, David Morales-Alamo, Björn Ekblom, Jose A. L. Calbet, Hans-Christer Holmberg, Robert Boushel

**Affiliations:** ^1^Åstrand Laboratory, The Swedish School of Sport and Health Sciences, Stockholm, Sweden; ^2^Department of Physiology and Pharmacology, Karolinska Institutet, Stockholm, Sweden; ^3^Department of Physical Education, University of Las Palmas de Gran Canaria, Las Palmas, Spain; ^4^Research Institute of Biomedical and Health Sciences (IUIBS), Las Palmas de Gran Canaria, Gran Canaria, Spain; ^5^School of Kinesiology, Faculty of Education, The University of British Columbia, Vancouver, BC, Canada; ^6^Swedish Winter Sports Research Centre, Department of Health Sciences, Mid Sweden University, Östersund, Sweden

**Keywords:** sexual dimorphism, mitochondria, endurance performance, mitochondrial function, skeletal muscle, OXPHOS

## Abstract

Sexual dimorphism is apparent in humans, however, to date no studies have investigated mitochondrial function focusing on intrinsic mitochondrial respiration (i.e., mitochondrial respiration for a given amount of mitochondrial protein) and mitochondrial oxygen affinity (p50_mito_) in relation to biological sex in human. A skeletal muscle biopsy was donated by nine active women, and ten men matched for maximal oxygen consumption (VO_2max_) and by nine endurance trained men. Intrinsic mitochondrial respiration, assessed in isolated mitochondria, was higher in women compared to men when activating complex I (CI_P_) and complex I+II (CI+II_P_) (*p* < 0.05), and was similar to trained men (CI_P_, *p* = 0.053; CI+II_P_, *p* = 0.066). Proton leak and p50_mito_ were higher in women compared to men independent of VO_2max_. In conclusion, significant novel differences in mitochondrial oxidative function, intrinsic mitochondrial respiration and p50_mito_ exist between women and men. These findings may represent an adaptation in the oxygen cascade in women to optimize muscle oxygen uptake to compensate for a lower oxygen delivery during exercise.

## Introduction

Sexual dimorphism of anatomical and physiological characteristics is apparent in humans as the result of differential gene expression between males and females (Rigby and Kulathinal, 2015). Importantly, the phenotypic differences between women and men have repercussions on life expectancy (Seifarth et al., 2012), disease occurrence and aging (Popkov et al., 2015; Tower, 2017). Nevertheless, women are significantly less studied than men in all disciplines of medical research (Costello et al., 2014) which highlights the need for further research in this field (Clayton and Collins, 2014).

Since the seminal work of Holloszy (1967) it has been shown that endurance exercise training enhances muscle oxidative capacity, through upregulation of both the activity and content of several mitochondrial enzymes, adaptations of which are quickly reversed by detraining (Holloszy and Booth, 1976; Henriksson and Reitman, 1977; Granata et al., 2016). To date, most studies have been conducted in men, without attention to potential physiological differences between sexes (Della Torre and Maggi, 2017). There are known sex-specific metabolic differences in skeletal muscle. For example, it has been shown that women oxidize more fatty acids and less carbohydrates than men at the same relative exercise workload (Tarnopolsky et al., 1990; Horton et al., 1998) potentially due to a higher mitochondrial content (Montero et al., 2018), higher baseline lipoprotein lipase levels (Skelly et al., 2017), estrogen (Ruby et al., 1997), and glycerol (Roepstorff et al., 2002) compared to men with similar cardiorespiratory fitness. Transcription and translation of proteins involved in muscle lipid metabolism are also sex dependent (Kiens et al., 2004) with women possessing a higher number of intramyocellular lipid droplets compared to men (Tarnopolsky et al., 2007). Likewise, a bout of endurance exercise has been shown to acutely increase the mRNA content of citrate synthase (CS) and β-hydroxyacyl-CoA dehydrogenase (HAD) to a larger extent in women compared to men (Roepstorff et al., 2005). However, others have reported similar gene expression in women and men following a bout of sprint interval exercise (Scalzo et al., 2014; Skelly et al., 2017) and similar signaling responses (Fuentes et al., 2012) with the exception of the exercise response of mRNA content of glucose transport 4, lipoprotein lipase and Atrogin-1, which have been shown to be sex-specific (Skelly et al., 2017). 5′AMP-activated protein kinase (AMPK), which acts as a cellular energy sensor, responds more robustly in men than women following similar relative energetic stress suggesting that women better preserve muscle cellular homeostasis compared to men following an exercise bout (Roepstorff et al., 2006). While the activity of mitochondrial enzymes such as 3-beta-Hydroxyacyl CoA dehydrogenase, complex II-III, cytochrome c oxidase, and CS activity are similarly improved by endurance training in men and women (McKenzie et al., 2000; Carter et al., 2001; Skelly et al., 2017), a greater muscle protein synthesis and mitochondrial biogenesis has been reported in men compared to women following sprint interval training (Scalzo et al., 2014). Although animal models have consistently shown the superiority of mitochondrial function of females compare to males (e.g., higher oxidative capacity, lower ROS production, higher fatty acid utilization, lower ADP-stimulated respiration in female compared to male) (Ventura-Clapier et al., 2017), data in humans is limited.

One study compared oxidative phosphorylation in the isolated mitochondrial preparation from gastrocnemius muscle finding no differences between men and women (Thompson et al., 2013). However, men and women were not matched according to any physiological criteria and all subjects had endothelial dysfunction. These two factors may have influenced the study outcomes. Two recent studies using the permeabilized fiber technique found that women possess similar maximal oxidative phosphorylation capacity per unit muscle mass (Miotto et al., 2018; Montero et al., 2018), but lower ADP sensitivity and different substrate sensitivity independent of maximal oxidative phosphorylation and mitochondrial content or protein level compared to men.

Recent findings in *Drosophila melanogaster* indicate that male mitochondria harbor a higher mutational load due to the maternal transmission of mitochondrial genes (Innocenti et al., 2011) which impact mitochondrial respiration and intrinsic mitochondrial oxidative phosphorylation capacity (i.e., mitochondrial respiration for a given amount of mitochondrial protein). Intrinsic mitochondrial function has not been directly assessed and compared in women and men with similar fitness level. A critical mitochondrial property that is less often investigated is the mitochondrial affinity for oxygen defined as the oxygen tension were mitochondrial respiration is 50% of its maximum (p50_mito_). Seminal work by Gnaiger et al. (1995) demonstrated the interdependence between mitochondrial efficiency and p50_mito_; mitochondrial coupling and thus mitochondrial efficiency is directly related to p50_mito_.

Extending these findings to the whole-body level, it has been shown that a low O_2_ affinity (high p50_mito_), is associated with a low basal metabolic rate (Schiffer et al., 2016) and high aerobic efficiency during exercise (Larsen et al., 2011). Furthermore, it has been recently shown that a high mitochondrial O_2_ affinity enhances O_2_ extraction during exercise (Cardinale et al., 2018b). The mechanistic basis for the association between p50_mito_ and efficiency can be explained by the presence of different isoforms of cytochrome c oxidase subunit IV where isoform IV-1 is associated with high affinity for oxygen but a low efficiency, whereas isoform IV-2 has a low affinity for oxygen but high efficiency (Schiffer et al., 2016). An alternative explanation based on thermodynamic processes indicates that a trade-off exists between the catalytic efficiency of an enzymatic process and its substrate affinity (Stucki et al., 1983). In other words, metabolic processes cannot achieve maximum efficiency and maximum power production simultaneously. Since it is well established that women have a higher efficiency during exercise (Åstrand et al., 2003; Fares et al., 2017) and lower basal metabolic rate per unit body mass (Benedict and Emmes, 1915), a logical premise is that women should have a higher p50_mito_ than men. Lowering the basal metabolic rate and increasing exercise efficiency with a higher p50_mito_ would be advantageous in women during periods of low energy availability. However, p50_mito_ in human skeletal muscle has only been reported in four studies to date (Larsen et al., 2011; Boushel et al., 2015; Schiffer et al., 2016; Cardinale et al., 2018b) and any difference between sexes have not yet been reported.

In line with the US National Institutes of Health call on integrating sex as a biological variable into animal and human research (Clayton and Collins, 2014), the aim of this paper was to examine if sex-based differences exist in mass-specific mitochondrial oxidative phosphorylation, intrinsic mitochondrial capacity (i.e., mitochondrial quality; mitochondrial respiration for a given amount of mitochondrial protein) and p50_mito_. To address this question, we compared skeletal muscle mitochondrial function in women and men with a similar maximal oxygen uptake (VO_2max_), and men with a higher VO_2max_. We hypothesized that women would show similar mass-specific mitochondrial oxidative phosphorylation capacity, but a higher intrinsic mitochondrial respiration and higher p50_mito_ compared to men with similar VO_2max_. We further hypothesized that women would have lower mass-specific and intrinsic mitochondrial respiration as well as a lower p50_mito_ compared to men with higher VO_2max_.

## Materials and Methods

### Subjects

A group of active women (Women *n* = 9) was matched to a group of men (Men *n* = 10) with similar maximal oxygen consumption (VO_2max_) and compared for all measured outcomes. The group of women all exercised regularly mainly running and cycling three or four times each week and some women were involved in competitive endurance events. All of the women tested in this study had a menstrual cycle of normal length (self-reported) and did not use hormonal contraceptives during and 3 months prior to the study period. It has been demonstrated previously that significant inter-and intra-subject variability exists with respect to ovarian hormone levels throughout the menstrual cycle that has effects on whole-body metabolism (Macnutt et al., 2012). As such, we tested women at random points throughout their menstrual cycle. The group of men all exercised recreationally (running, cycling, etc.) two or three times each week but none trained regularly for any particular sporting event. Further comparisons were made including a third group of endurance trained men (Trained men *n* = 9) with higher VO_2max_ compared to both the women and men groups. This third group was comprised of trained cyclists who trained about 7–9 times each week, 60–300 min per training session over the past 5–10 years and all competed in endurance events. Subject characteristics are presented in **Table 1**. All subjects were determined healthy based on a health screening survey and were informed about possible risks and discomfort involved before giving their written consent to participate in this study. The study was undertaken according to the Declaration of Helsinki and was approved by the Regional Ethical Review Board in Stockholm and in Umeå, Sweden.

**Table 1:** Subject’s characteristics.

Group	*N*	Sex	Age	Body mass (kg)	Height (cm)	VO_2max_ (L min^-1^)	VO_2max_ (mL min^-1^ kg^-1^)
Women	9	Female	28.56 ± 2.65	63.26 ± 8.74	168.24 ± 8.45	3.21 ± 0.37	50.98 ± 4.08
Men	10	Male	28.01 ± 4.82	74.37 ± 4.95*	180.93 ± 5.98*	3.60 ± 0.43	49.27 ± 4.07
Trained men	9	Male	33.67 ± 7.43	73.51 ± 8.94*	181.67 ± 8.93*	4.91 ± 0.51*	67.07 ± 3.46*


*^∗^Significant difference from women group.*

### Systemic Oxygen Consumption

A graded incremental exercise test until volitional exhaustion on a cycle ergometer was used to determine VO_2max_ except for three women who performed a graded incremental exercise on a treadmill. These three women were later tested both on a cycle ergometer and on a treadmill for VO_2max_ assessment and their VO_2max_ was not different between exercise modes. VO_2max_ leveling-off criteria was applied (i.e., a VO_2_ plateau, followed by exercise cessation or decrease of VO_2_ at higher work rates, with an RER > 1.10). The maximal power output (W_max_) pedaled during the graded incremental exercise test was taken as individual cycle work capacity. O_2_ consumption was measured with a metabolic cart with a mixing chamber (Oxycon Pro, Jaeger GmbH, Germany), calibrated prior to each test according to the manufacturer’s instructions, with high-grade calibration gases (Air Liquide, Sweden). Respiratory variables were measured and averaged every 10 s. The highest 30 s averaged VO_2_ recorded was taken as the VO_2max_.

### Muscle Biopsy Sampling

In a resting state after a minimum of 48h without any strenuous physical activity, a muscle sample was obtained from the middle portion of the *vastus lateralis* muscle at a depth of 2–3 cm, about one-third of the distance from the upper margin of the patella to the anterior superior iliac spine. After local anesthesia (2–4 ml Carbocaine 20 mg ml^-1^; Astra Zeneca, Södertälje, Sweden) an incision (0.5–1 cm) was made through the skin and fascia and a muscle sample (50–100 mg) was obtained either with the Weil-Blakesley conchotome technique or modified Bergström needle with suction (Ekblom, 2017). A part of the specimen (∼50 mg) was rapidly placed in ice-cold mitochondrial isolation medium (Sucrose 100 mM, KCl 100 mM, Tris-HCl 50 mM, KH_2_PO_4_ 1 mM, EGTA 100 μM, BSA 0.1%; final pH was set to 7.4) followed by mitochondria isolation.

### Isolation of Mitochondria

Mitochondrial isolation was conducted as published (Tonkonogi and Sahlin, 1997) but with a minor modification as described by Larsen et al. (2011). Briefly, the muscle biopsy was first weighed and then cut in ice-cold isolation medium (Sucrose 100 mM, KCl 100 mM, Tris-HCl 50 mM, KH_2_PO_4_ 1 mM, EGTA 100 μM, BSA 0.1%; final pH was set to 7.4) (Gnaiger and Kuznetsov, 2002). One milliliter of isolation medium containing 0.2 mg ml^-1^ bacterial protease was then added to the homogenate and then transferred to a glass jacket connected to an ice-cold bath pump for further homogenization with a hand held electrically driven drill (80 rpm). The final homogenate was centrifuged at 700 *g* at 4°C for 10 min. The supernatant was again centrifuged at 10000 *g* at 4°C and the resultant mitochondrial pellet was re-suspended in the same medium. The Eppendorf was then centrifuged at 7000 *g* for 5 min and the pellet was dissolved in 0.6 μl preservation medium (EGTA 0.5 mM, MgCl_2_ 6H_2_O 3 mM, K-lactobionate 60 mM, Taurine 20 mM, KH_2_PO_4_ 10 mM, HEPES 20 mM, Sucrose 110 mM, BSA 1 g L^-1^ Histidine 20 mM, Vitamin E succinate 20 μM, Glutathione 3 mM, Leupeptine 1 μM, Glutamate 2 mM, Malate 2 mM, Mg-ATP 2 mM) (Gnaiger and Kuznetsov, 2002) per initial wet weight of the sample from where mitochondria were isolated.

Mitochondrial respiration was performed in a two-channel high resolution respirometer (Oroboros Oxygraph, Paar, Graz, Austria). Data sampling was set for 1 s intervals and averaged over 40 s. Each experiment was run twice and the O_2_ flux of the two chambers were then averaged. MiR05 medium (EGTA 0.5 mM, MgCl_2_.6H_2_O 3 mM, K-lactobionate 60 mM, Taurine 20 mM, KH_2_P0_4_ 10 mM, HEPES 20 mM, Sucrose 110 mM, BSA 1 g L^-1^) was used to assess respiration. All experiments were performed at 37°C. O_2_ consumption and zero-drift of the O_2_ electrode were calculated using DatLab 5.2 software (Oroboros, Paar, Graz, Austria).

### Mitochondrial Respiratory Protocols

Mitochondrial respiration was measured with the titration of substrates into the chambers to assess leak respiration (L) with malate (2 mM) and pyruvate (5 mM) in the absence of adenylates; complex I respiration (CI_P_) with addition of saturating ADP (5 mM); state 4 respiration [respiration after state 3 (Chance and Williams, 1955) when ADP is phosphorylated to ATP], followed by convergent complex I+II linked ADP-stimulated maximal respiration (CI+II_P_) with addition of succinate (10 mM). In three women the substrate titration protocol used for assessment of leak respiration was obtained in the absence of adenylates titrating palmitoylcarnitine (0.2 mM) in addition to malate and pyruvate, and CI+II_P_ was obtained titrating glutamate (10 mM) in addition to succinate. The respiration data from these three women are included in the current figures. Based on several experiments conducted in our laboratory we have found no effect of the slightly different substrate combinations on the results we report here. We have done a separate analysis of the current data excluding these data points and we detect the same statistical differences between groups. Mitochondrial respiration rates (pmol s^-1^) were normalized for mitochondrial suspension protein levels (pmol s^-1^ μg^-1^) in the isolated mitochondria preparation using the Pierce 660 nm protein assay (Thermo Fisher Scientific) to obtain the intrinsic mitochondrial respiration and additionally normalized for initial wet weight of the muscle sample (pmol s^-1^ mg^-1^) to obtain mass-specific mitochondrial respiration. The latter mass-specific respiration expressed as pmol s^-1^ mg^-1^ initial wet weight are a calculation and as such are much lower and not comparable to those reported from direct respiration measures in permeabilized fibers (Cardinale et al., 2018a). The p50_mito_ in isolated mitochondria was defined as the O_2_ tension where mitochondrial respiration proceeds at 50% of the maximum rate in presence of saturating ADP concentrations. p50_mito_ was determined by smoothing the exponential O_2_ flux decay corrected for time-constant, background correction, and zero oxygen calibration using DatLab 2 software (Oroboros Oxygraph, Paar, Graz, Austria).

### Statistics

Results are presented as mean ± SD. The data were initially tested for normal distribution and equal variance using the Shapiro–Wilk test of normality and Q-Q plots. Not-normally distributed parameters where log transformed. One-way analysis of variance was used to assess the between group differences for the normal distributed data. A *p* < 0.05 was considered significant. Statistical analyses were carried out using SPSS statistical software version 21 (SPSS Inc., Chicago, IL, United States).

## Results

### Intrinsic Mitochondrial Respiration Differs Between Men and Women With Similar VO_2max_

**FIGURE 1:**
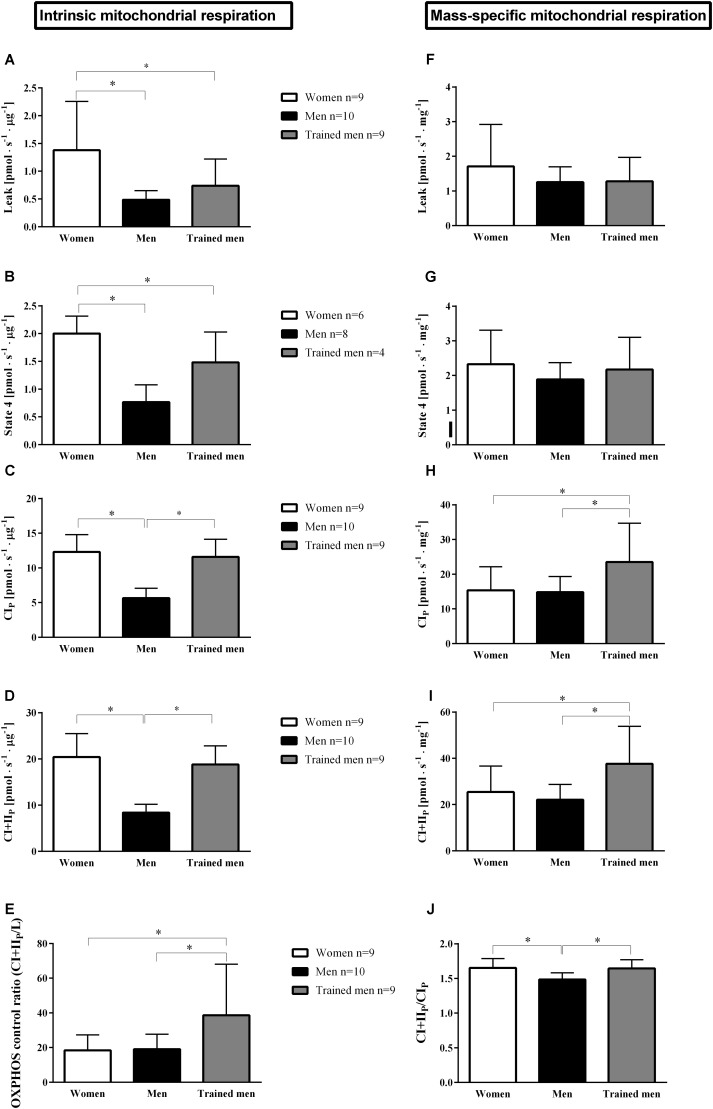
Mean ± SD of mitochondrial respiration rates normalized by protein levels of mitochondrial suspension (pmol s^-1^ μg protein^-1^; intrinsic mitochondrial oxidative respiration) and by the initial muscle wet weight (pmol s^-1^ mg^-1^; mass-specific mitochondrial respiration) in women, men, and trained men groups for: **A**,**F**, leak respiration is the respiratory rate in the presence of substrates without addition of adenylates; **B**,**G**, state 4 is the respiratory rate when ADP is phosphorylated maximally to ATP; **C,H**, complex I_P_ is the maximum ADP stimulated respiration rate in the presence of complex I substrates; **D,I**, complex I+II_P_ is similar to **C,H** but with added convergent electron flux through complex II by adding succinate (CI+II_P_); **E**, maximal oxidative phosphorylation capacity (OXPHOS) control ratio (CI+II_P_/Leak) indicates the limitation of OXPHOS by the phosphorylation system; **J**, CI+II_P_/CI_P_ indicates the contribution of CII respiration to maximal respiration. ^∗^*p* < 0.05 between groups.

Mitochondrial oxidative phosphorylation capacity depends on mitochondrial content and quality (i.e., intrinsic mitochondrial respiration). To test the hypothesis that women possess a higher intrinsic mitochondrial respiration compared to men with similar VO_2max_, we measured maximal ADP-stimulated mitochondrial respiration in isolated mitochondria. Mitochondrial respiration when providing electron donors specific to either complex I (CI_P_) or complex I + II (CI+II_P_) normalized for mitochondrial protein concentration was higher in women (*p* < 0.05, **Figures 1C,D**) than in men with similar VO_2max_. We therefore sought to test if the intrinsic mitochondrial respiration in the women would be similar to men with a higher VO_2max_ (i.e., trained men group). Interestingly, mitochondrial respiration activating CI_P_ and CI+II_P_ (**Figures 1C,D**) did not differ between women with a VO_2max_ of 51.0 ± 4.1 mL O_2_min^-1^ kg^-1^ and trained men whose VO_2max_ was 67.1 ± 3.4 mL O_2_min^-1^ kg^-1^.

### Mass-Specific Mitochondrial Respiration

Having established that intrinsic mitochondrial respiration was higher in women than in men with similar VO_2max_ we tested if this factor was balanced out by a higher mitochondrial content in men compared to women. Therefore, we related the mitochondrial respiratory parameters to initial wet weight of the muscle (see section “Materials and Methods”) used in the mitochondrial isolation procedure (**Figures 1F–I**). Mass-specific mitochondrial respiration did not differ between women and men with similar VO_2max_. When compared to the trained men with higher VO_2_ max, women had lower mass-specific mitochondrial respiration. These results would indicate that women possess a higher intrinsic mitochondrial respiration, and a lower mitochondrial abundance resulting in similar mitochondrial respiration per wet weight for a given whole body aerobic capacity compared to men or that women do not need to increase their mitochondrial content to the same extent as men to have similar respiration per wet weight muscle since women possess higher intrinsic mitochondrial respiration.

### Higher Intrinsic Proton Leak in Women Than in Men

Leak respiration (mitochondrial respiration in the absence of adenylates) has been used as a proxy for passive proton leak over the inner mitochondrial membrane. It has been estimated that proton leakage accounts for a major part of the basal metabolic rate (Porter and Brand, 1993). Since women have lower basal metabolic rate than men it is logical to assume that women should have lower mitochondrial leak respiration than men. Surprisingly, intrinsic leak respiration was significantly higher (*p* < 0.05) in women compared to men independent of VO_2max_ (**Figure 1A**). Consistent with this finding, intrinsic state 4 respiration (respiratory rate obtained in isolated mitochondria when ADP has been phosphorylated to ATP) was higher in women than in men independent of VO_2max_ (**Figure 1B**). However, when leak respiration and state 4 respiration were related to initial wet weight, no significant differences were found between sexes or groups.

### The Relative Contribution of Complex II to the Total Electron Flux Is Higher in Women Compared to Men

Women and men of the same VO_2max_ had similar respiratory control ratio (*p* > 0.05), measured as the ratio of leak respiration to maximal oxidative phosphorylation rate (CI+II_P_/L), but respiratory control ratio was lower in women compared to the trained men (*p* < 0.05) (**Figure 1E**). When complex II is activated by addition of succinate, respiration increases compared to when respiration is only supported by complex I. The relative ratio of this increase is a measure of the relative contribution of complex II to the total electron flux and we found this ratio to be higher in women compared to men matched for VO_2max_, but similar to trained men (**Figure 1J**).

### p50_mito_ Differs Between Sexes

**FIGURE 2:**
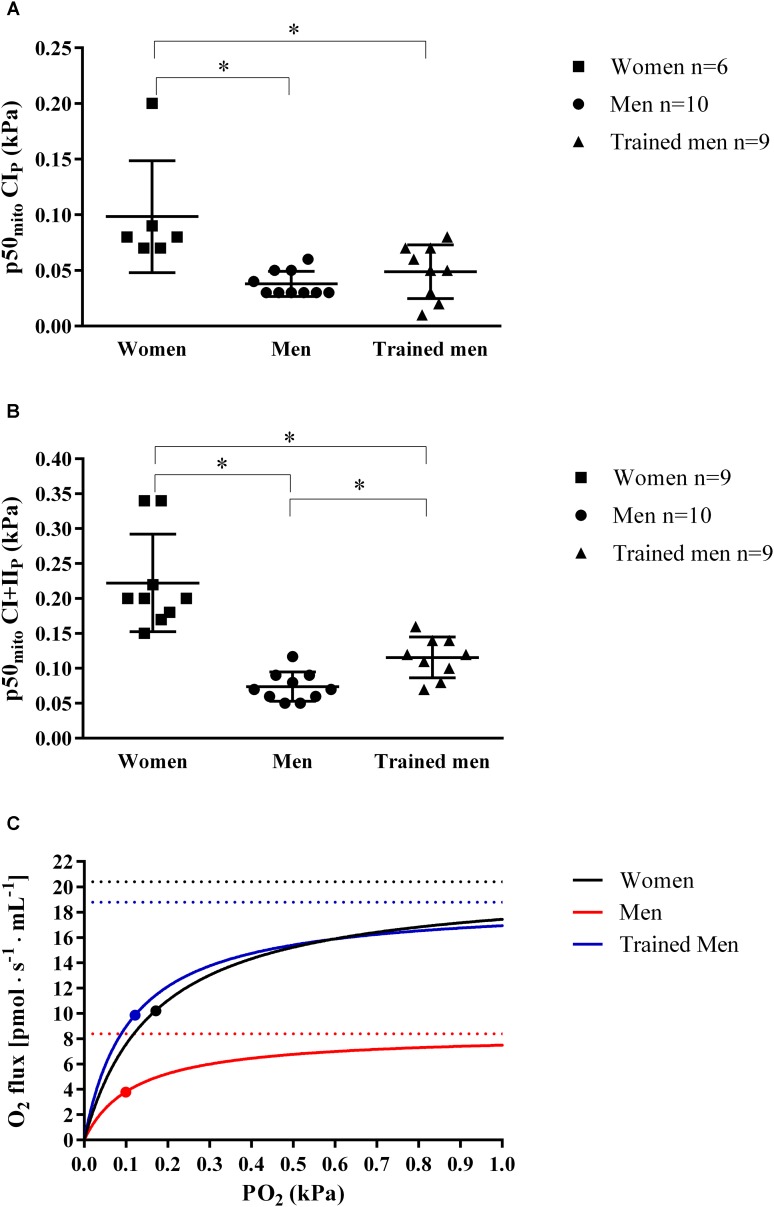
**(A)** Individual and mean ± SD values of *ex vivo* mitochondrial p50 (p50_mito_; kPa) at maximal ADP-induced activation measured in isolated mitochondria when activating complex I (CI_P_) for women, men, and trained men groups. **(B)** Individual and mean ± SD values of *ex vivo* p50_mito_ as shown in **A**, but with complex I+II-linked substrate state (CI+II_P_) for women, men, and trained men groups. ^∗^*p* < 0.05 between groups. **(C)** Hyperbolic curves showing the relation between PO_2_ (kPa) and mitochondrial O_2_ flux [pmol s^-1^ mL^-1^]. Curves were obtained inserting into the following equation [VO_2_ = (CI+II_P_ PO_2_)⋅(PO_2_ + p50_mito_)^-1^] continuous PO_2_ values, the *ex vivo* p50_mito_ (graphically indicated with a dot) and CI+II_P_ (graphically indicated with a dashed lines) mean values measured in this study in women, men, and trained men groups. This figure shows the role of mitochondrial p50 for muscle oxygen consumption since for a given intracellular PO_2_, a lower p50_mito_ would result in a higher tissue O_2_ consumption [VO_2_ = CI+II_P_⋅PO_2_ (PO_2_ + p50_mito_)^-1^] and vice versa. The superior intrinsic mitochondrial respiration in women compared to men with similar VO_2max_ shown in this study may be an important physiological adaptation that compensates for the higher mitochondrial p50 allowing a higher O_2_ extraction peripherally.

The discovery that women have higher mitochondrial intrinsic capacity (i.e., O_2_ flux per mitochondrial protein) than men with similar VO_2max_ led us to test if this difference was linked to difference in mitochondrial O_2_ affinity (p50_mito_). We measured p50_mito_ by titrating substrates which activate CI_P_ and CI+II_P_ with saturating ADP concentrations. In women, the p50_mito_ with complex I substrates (0.10 ± 0.05 kPa) and with complex I+II substrates (0.22 ± 0.07 kPa) was significantly higher (*p* < 0.05) than in men with similar VO_2max_ (p50_mito_ with CI_P_ = 0.04 ± 0.01 kPa, p50_mito_ with CI+II_P_ = 0.07 ± 0.02 kPa), and also higher compared to trained men with the higher VO_2max_ (p50_mito_ with CI_P_ = 0.05 ± 0.02 kPa, p50_mito_ with CI+II_P_ = 0.12 ± 0.03 kPa) (**Figures 2A–C**).

### Cycle Work Capacity

**FIGURE 3:**
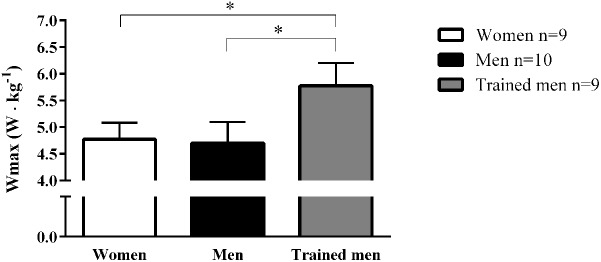
Mean ± SD values of maximal cycling power output (W_max_; W kg^-1^) achieved on a gradual incremental exercise test for women, men, and trained men groups. ^∗^*p* < 0.05 between groups.

It is well known that endurance trained athletes have both high cardiac output and mitochondrial respiratory capacity. It is well accepted that cardiac output is a major limiting factor in the oxygen cascade (Bassett and Howley, 2000; Saltin and Calbet, 2006). A greater mitochondrial capacity has instead been hypothesized to be more important for endurance performance and physical work capacity (Gollnick and Saltin, 1982). Since our subjects were matched for VO_2max_ we wanted to test if cycling work capacity differed between groups due to the observed differences in mitochondrial characteristics. The maximal power output pedaled during the incremental cycling ergometer test did not differ between women and men for comparable VO_2max_ but was significantly lower compared to the trained men group with the higher VO_2max_ when normalized per individual body mass (**Figure 3**).

**FIGURE 4:**
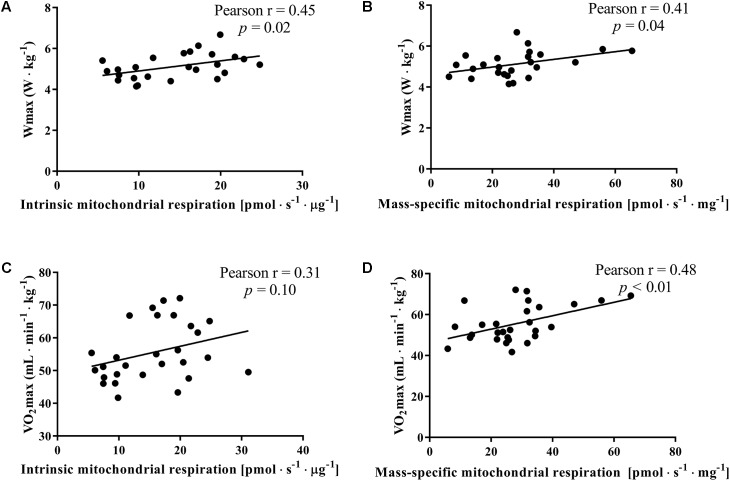
Correlation between the maximal mitochondrial oxidative phosphorylation (State 3 complex I+II_P_), maximal cycling power output (W_max_) achieved on a gradual incremental exercise test and maximal oxygen consumption (VO_2max_) for the whole group of subjects. Maximal mitochondrial oxidative phosphorylation was normalized for mitochondrial protein content (intrinsic mitochondrial respiration; **A,B**) and for initial wet weight (mass-specific mitochondrial respiration; **C**,**D**).

In our subjects, the maximal power output pedaled during the incremental cycling was significantly correlated to the intrinsic and mass-specific mitochondrial respiration activating CI+II_P_ and as expected VO_2max_ was better correlated to mass-specific mitochondrial respiration than intrinsic mitochondrial respiration (**Figure 4**).

## Discussion

This study presents new insights on physiological sexual dimorphism in human skeletal muscle mitochondria. Here for the first time, we provide experimental evidence in humans that intrinsic and leak respiration are higher in women compared to men with similar mass-specific mitochondrial respiratory capacity. Furthermore, mitochondrial oxygen affinity is lower in women compared to men for a similar VO_2max_. When compared to endurance-trained men, women have similar intrinsic mitochondrial respiration but lower mass-specific respiration, lower mitochondrial oxygen affinity, higher intrinsic proton leak, and lower cycle work capacity.

### Mitochondrial Quality in Women and Muscle Metabolism

Strong sexual dimorphism has been shown in rodent models with females exhibiting superior structural and functional mitochondria in different organs compared to males (Ventura-Clapier et al., 2017). It appears that the part of the genome regulating mitochondrial function is optimized in women since mitochondrial DNA is almost exclusively maternally inherited (Tower, 2015). The higher intrinsic mitochondrial respiration in women could be a physiological strategy to increase energy yield from fat oxidation which is usually found to be higher in women than in men during exercise at a given relative work rate (Tarnopolsky et al., 1990; Roepstorff et al., 2006). AMPK has been implicated in the regulation of fatty acid uptake, handling, and oxidation (Thomson and Winder, 2009; O’Neill et al., 2013) as well as mitochondrial biogenesis via direct phosphorylation of peroxisome proliferator activated receptor c co-activator-1a (Norrbom et al., 2011) (the transcriptional regulator of genes involved in oxidative metabolism). Therefore, a potential mechanism for the higher fat oxidation in women could reside in a greater AMPK signaling. However, no sex differences have been found in resting AMPK, while a lower AMPK activation following exercise has been reported in women compared to men (Roepstorff et al., 2006) indicating that AMPK signaling may not the be key regulator of fat oxidation during prolonged exercise in women and not differentially regulating intrinsic mitochondrial respiration in women and men. Furthermore, in a well-controlled study design, a 3h exercise bout on a cycle ergometer performed at a work intensity of 60–65% of VO_2max_ (work intensity at which fat oxidation is expected to be maximized), resulted in no difference in fat oxidation between well-trained women and men, but a higher carbohydrate oxidation in well-trained men (Zehnder et al., 2005). It could be argued that if women have enhanced capacity for fat oxidation during exercise or possess any other muscle metabolic advantage this would give them an edge during long endurance events. However, racing times in endurance events ranging between 1500 m to marathon is ∼11% lower (faster) in men compared to women (Sparling et al., 1998). This performance gap between women and men increases to about 20% in ultra-events (mainly running and cycling events) (Knechtle et al., 2014; Zingg et al., 2014). There is some exception in swimming, especially open-water events, where women perform equally or better compared to men (Knechtle et al., 2015) likely due to the increased buoyancy in women attributed to the caudally located higher percentage of fat mass (McLean and Hinrichs, 1998). These differences are likely not explained by differences in running economy between women and men (Daniels et al., 1977; Davies and Thompson, 1979) although conflicting results have been reported (Bransford and Howley, 1977; Bhambhani and Singh, 1985).

### VO_2max_ Is a Stronger Determinant of Work Capacity Than Mitochondrial Capacity

An interesting observation is that the maximal work load during the graded incremental exercise test normalized for individual body mass, which highly relates to cycling endurance performance (Faria et al., 2005), did not differ between women and men with comparable VO_2max_ despite a ∼143% higher intrinsic mitochondrial respiration in women. This would indicate that VO_2max_ is a stronger determinant of work capacity than intrinsic mitochondrial respiration (**Figure 4**).

### The Role of Skeletal Mitochondrial Oxidative Capacity and p50_mito_ in Regulating Oxygen Consumption

The higher skeletal mitochondrial respiratory capacity in women compared to men relative to VO_2max_ has important implications for our understanding of regulatory factors in the O_2_ cascade during exercise. Based on conservation of mass, a high mitochondrial respiratory capacity relative to O_2_ delivery indicates that at VO_2max_, mitochondria respire at a substantially lower relative mitochondrial respiratory capacity (Boushel et al., 2015). The higher maximal ADP-stimulated intrinsic mitochondrial respiration rate in women compared to men of similar VO_2max_ indicates that during exercise, mitochondria isolated from women respire at a *lower* relative rate. In the present study we show that the *in vitro* maximally ADP-stimulated p50_mito_ was higher in women (O_2_ affinity was lower) compared to men. However, the p50_mito_ *in vivo* is also a function of the relative activation of mitochondria (at what fraction of the maximal respiratory rate the mitochondria respires at whole-body VO_2max_ intensity). Since women show both a higher intrinsic mitochondrial respiration and a higher p50_mito_, these two variables may balance out *in vivo*. A lower relative activation of mitochondria at VO_2max_ *in vivo* in women would also lower the p50 (Cardinale et al., 2018b). As recently demonstrated both mitochondrial capacity and p50_mito_ have important implications for muscle oxygen consumption since for a given intracellular PO_2_, lower p50_mito_ would result in a higher tissue O_2_ consumption [VO_2_ = CI+II_P_ PO_2_ (PO_2_ + p50_mito_)^-1^] (Cardinale et al., 2018b). The higher mitochondrial capacity in women may thus be an important physiological adaptation in women to permit a higher O_2_ extraction peripherally to counteract the more centrally mediated limitations of a higher work of breathing (Dominelli et al., 2013, 2017) and lower O_2_ carrying capacity of blood (Murphy, 2014). The lower relative activation of mitochondria in women during exercise could also preserve fat oxidation capacity, maintain mitochondrial efficiency, and lower ROS production.

### Higher Intrinsic Proton Leak in Women Than in Men

Our results indicate an unexpected overall higher intrinsic proton leakage in women compared to men. This can be another strategy to lower ROS production by reducing the mitochondrial membrane potential and could also be linked to the longer life-span in women since the aging process has been linked to ROS-production (Finkel and Holbrook, 2000). Uncoupling protein-3 (Gong et al., 1997) and ANT (Andreyev et al., 1989; Azzu et al., 2008) are thought to dissipate energy as heat and affecting ATP production which could be responsible for the higher intrinsic proton leak in women compared to men. However, the higher proton leak compared to men did not compromise mitochondrial coupling efficiency which was similar between women and men with similar VO_2max_ (**Figure 1E**). Interestingly, the contribution of complex II of the electron transfer system to the total coupled mitochondrial respiratory capacity (**Figure 1J**) was greater in women than in men with similar VO_2max_. This would indicate a functional difference of specific proteins of the electron transfer system between women and men.

These findings raise the important question of whether the observed differences between women and men are a true sex difference as a consequence of evolutionary pressure (Della Torre and Maggi, 2017) or are the result of environmental factors such as the response to exercise training or other factors such as nutrition and lifestyle.

### Mitochondrial Quality: Born or Made?

Both high-volume low-intensity endurance exercise (Daussin et al., 2008; Vincent et al., 2015) and low-volume high-intensity endurance exercise (Granata et al., 2015) are strategies capable of improving mitochondrial oxidative capacity (Holloszy, 1967; Holloszy and Booth, 1976). However, cross-sectional data indicate that high-intensity training is a key factor to stimulate higher mitochondrial respiration whereas training volume is to a greater extent linked to change in mitochondrial content (i.e., higher CS activity) (Bishop et al., 2014; Vigelsø et al., 2014). Nevertheless, the improvement in mass-specific mitochondrial respiration usually reported at the end of an endurance exercise intervention usually disappears when mitochondrial respiration is normalized to a mitochondrial parameter such as mitochondrial protein content or CS activity (Granata et al., 2016; MacInnis et al., 2016). Accordingly, it is unclear which training regimen stimulates intrinsic mitochondrial respiration and which mitochondrial components underlie increased intrinsic mitochondrial respiration (Bartlett et al., 2017). A plausible locus of regulation is the mitochondrial cristae, which until recently has been thought to be of constant density relative to mitochondrial volume among individuals; however, differences between active individuals and elite athletes was recently discovered (Nielsen et al., 2016). It has been proposed that endurance exercise induces mitochondrial biogenesis and leads to the development of new mitochondria which at the initial stage become enlarged, followed by an increase in length (Glancy et al., 2015; Lundby and Jacobs, 2015), and lastly an increase in mitochondrial cristae density, since further increase in mitochondrial content would impair muscle contractile function (Nielsen et al., 2016). With this background, it is possible that women in our study may have had higher mitochondrial cristae density compared to men with similar VO_2max_.

In addition to the higher mitochondrial cristae density hypothesis, a second plausible explanation for the superior intrinsic mitochondrial respiration in women compared to men could be a higher abundance of supercomplexes and respirasomes (Schagger and Pfeiffer, 2000; Lobo-Jarne and Ugalde, 2018) in women. Supercomplexes are electron transport system proteins aggregated in a supermolecular assemblies which more rarely are constituted of all the required proteins to transfer electrons from NADH to molecular oxygen, termed respirasomes. The morphology of these supercomplexes is optimized to increase the mitochondrial catalytic efficiency (Bianchi et al., 2004) and their abundance can be altered by exercise training (Greggio et al., 2016).

Generally, women possess higher % body fat than men and therefore it can be speculated that the superior intrinsic mitochondrial respiration in women compared to men with similar VO_2max_ in our study is the result of a chronic exposure of higher O_2_ delivery per lean muscle mass in women than in men. In support of this argument, it has been shown that mitochondria respire close to their maximal capacity when the exercised muscle is highly perfused such as in the case of one-legged knee exercise (Blomstrand et al., 2011). Furthermore, peripheral adaptations of skeletal muscle following one-legged cycling is greatly enhanced compared to double-leg cycling where lower O_2_ delivery per active muscle mass occur (Abbiss et al., 2011). Unfortunately, body composition assessment was not systematically measured in this study, therefore we are unable to present VO_2max_ scaled per lean muscle mass. However, it is reasonable to assume the group of women had a higher percentage fat mass compared to trained men, and therefore women with a VO_2max_ of 51.0 ± 4.1 mL O_2_ min^-1^ kg^-1^ had a similar mitochondrial quality compared to the trained men whose VO_2max_ was 32% higher. In other words, it would not be expected that the women in this study had 32% higher body fat than the men with the higher VO_2_ max. This would indicate that O_2_ delivery per unit muscle mass should not account for the difference in mitochondrial quality observed in women and men recruited in this study.

### Study Limitations

This study is not without limitations. First, we could not report VO_2max_ scaled per lean muscle mass. Second, the research design did not control for diet between participants and menstrual cycle phase when testing women; thus, a greater variability in the measured outcomes may have been introduced by these factors. Nonetheless, these potential influences are unlikely to account for the robust magnitude of difference in intrinsic mitochondrial respiration and p50_mito_ between women and men. The strength of this study includes the assessment of intrinsic mitochondrial respiration and p50_mito_ which has not been previously reported when comparing women to men.

## Conclusion

This study provides evidence that women may possess a superior mitochondrial quality than men with equal cardiorespiratory fitness and endurance performance. Such a difference could be due to compensatory adaptations in women in the oxygen cascade (Della Torre and Maggi, 2017). Considering the greater life expectancy (Seifarth et al., 2012), and lower disease occurrence and aging (Popkov et al., 2015; Tower, 2017) in women than in men, the present findings suggest that the reserve of mitochondrial oxidative capacity (Boushel et al., 2011) may be even more physiologically important in men (Tower, 2017). Future studies should focus on the possible *in vivo* physiological effects of a higher intrinsic mitochondrial respiration, identifying which mitochondrial components underlie a higher intrinsic mitochondrial respiration (e.g., supercomplexes, mitochondrial cristae density) as well as the implications of upregulating intrinsic mitochondrial respiration in diseased populations. Additional factors such as diet, potential hormonal effects associated with the menstrual cycle are important questions for future study. Whether the higher intrinsic mitochondrial function in women represents a compensatory peripheral adaptation to low blood oxygen content also remains an interesting question for related disciplines.

## Author Contributions

DC, FL, and RB contributed to the conception of the study. All authors contributed to the data collection. DC analyzed, interpreted the data, and wrote the first draft of the manuscript which was reviewed by FL and RB. All authors read and approved the final manuscript. All persons designated as authors qualify for authorship, and all those who qualify for authorship are listed.

## Conflict of Interest Statement

The authors declare that the research was conducted in the absence of any commercial or financial relationships that could be construed as a potential conflict of interest.
